# Immunomodulatory and Antioxidant Activities of *Pinus sylvestris* L. Xylem Sap: Modulation of NK-92 Cell Receptors and Cytotoxic Function

**DOI:** 10.3390/molecules31203581

**Published:** 2026-10-09

**Authors:** Hasan Uğur Öncel, Işık Neslişah Korkut, Ahmet Beyatli, Ömer Faruk Bayrak, Serap Kuruca

**Affiliations:** 1 Department of Nutrition and Dietetics, Faculty of Health Sciences, Istanbul Gedik University, Istanbul 34913, Türkiye; ugur.oncel@gedik.edu.tr; 2 Department of Physiology, Faculty of Medicine, Istanbul University, Turgut Özal Millet Str., Istanbul 34093, Türkiye; neslisahkorkut@hotmail.com; 3 Department of Medicinal and Aromatic Plants, Hamidiye Vocational School of Health Services, University of Health Sciences, Istanbul 34668, Türkiye; 4 Department of Medical Genetics, Faculty of Medicine, Yeditepe University, Istanbul 34718, Türkiye; ofbayrak@yeditepe.edu.tr; 5 Department of Physiology, Faculty of Medicine, Istanbul Atlas University, Istanbul 34408, Türkiye

**Keywords:** *Pinus sylvestris*, xylem sap, LC-MS/MS, natural killer cells, immunomodulation, antioxidant activity

## Abstract

This study investigated the phytochemical profile, antioxidant capacity, and immunomodulatory properties of *Pinus sylvestris* xylem sap harvested from the Kastamonu region of Türkiye during the early spring active sap-flow period. Quantitative screening demonstrated significant polyphenolic density, with total phenolic (39.65 ± 2.12 mg GAE/g) and flavonoid contents (21.48 ± 3.11 mg Catechin/g) reflecting a high relative accumulation of mobile, hydrophilic flavonoids. Targeted LC-MS/MS analysis further revealed the presence of several phenolic and flavonoid-related compounds, with catechin as the predominant compound in the directly extracted sample, while epicatechin and epigallocatechin were among the major compounds detected following acid hydrolysis. The sap exhibited consistent, robust antioxidant activity across DPPH (43.52 ± 2.76 mg TE/g), ABTS (41.64 ± 2.17 mg TE/g), and FRAP (39.21 ± 1.95 mg TE/g) assays, indicating multi-pathway radical scavenging and electron-donating capacities in cell-free chemical models. While direct exposure yielded concentration-dependent cytotoxicity against tumor lines (IC50 values: 7.6–12.5 µg/mL), subtoxic conditioning (1 µg/mL) and substantially enhanced NK-92-mediated lysis of target cancer cells, most prominently against MCF-7 breast carcinoma. Flow cytometric analysis revealed that xylem sap treatment selectively upregulated surface expression of CD16 (from 0.00% to 15.93%) and CD57 on NK-92 cells without altering baseline CD56. Furthermore, sap stimulation triggered a concentration-dependent surge in IFN-γ production and elevated cytotoxic responses in lymphocytes isolated from both healthy controls (*n* = 5) and breast cancer patients (*n* = 5). These findings indicate that conifer xylem sap represents in vitro immunomodulatory properties and enhances NK-92 cell-mediated cytotoxic responses in cell culture models.

## 1. Introduction

From a phytochemical perspective, the bioactivity of *P. sylvestris* is largely driven by its complex array of secondary metabolites. Extensive research has indicated that tissues of the genus *Pinus* are highly enriched with phenolic compounds, particularly flavonoids, stilbenes (such as pinosylvin), and proanthocyanidins. These compounds serve as powerful natural antioxidants, acting via free-radical scavenging mechanisms and metal chelation to defend biological systems against oxidative stress [1]. Although previous studies have extensively characterized the volatile constituents of pine essential oils and the extractable phytochemicals of bark, wood, and needles, comparatively little attention has been devoted to the chemical composition of xylem sap. Unlike bark, needles, cones, and resin, which represent relatively distinct and chemically complex solid plant matrices, xylem sap is a dynamic vascular fluid involved in the long-distance transport of water, mineral nutrients, sugars, amino acids, signaling molecules, and other dissolved metabolites [2,3,4]. Studies of *P. sylvestris* xylem and related conifer matrices have identified phenolic acids, stilbenes, lignan-related compounds, and other secondary metabolites, although their occurrence and abundance can vary according to tissue type, developmental stage, environmental conditions, and sampling period [3,4]. Thus, findings from solid xylem or wood cannot necessarily be extrapolated directly to the composition of the liquid xylem sap. Consequently, its chemical composition and biological activities may differ from those of other pine-derived products [3]. This distinction is important because, despite the well-characterized phytochemistry of pine bark, needles, resin, and wood, the chemical composition and biological potential of the liquid vascular sap of *P. sylvestris* remain comparatively poorly investigated. In particular, evidence regarding its antioxidant and immunomodulatory properties is limited, and its potential effects on NK-cell phenotype and function have not been adequately characterized. This knowledge gap provided the rationale for investigating xylem sap as a distinct biological matrix in the present study. Xylem sap represents the principal long-distance transport medium in vascular plants, facilitating the movement of water, mineral nutrients, and a variety of dissolved organic compounds, including sugars, amino acids, and signaling molecules. Recent studies have demonstrated that xylem sap chemistry in conifer species exhibits considerable temporal variation and may reflect important physiological and adaptive processes within the plant [4].

In recent years, interest has grown regarding the capacity of plant-derived bioactive compounds to modulate immune function, particularly within innate immune pathways [5,6]. As critical components of the innate immune system, Natural Killer (NK) cells provide an essential first line of defense responsible for early viral control and tumor immune surveillance [7]. The activation and functional dynamics of NK cells are governed by a delicate balance of activating and inhibitory signaling pathways that dictate cytotoxicity and the secretion of immunoregulatory cytokines, notably interferon-gamma (IFN-γ) [8]. Specific phenolic compounds, including flavonoids such as apigenin, luteolin, and quercetin, have been shown to directly modulate cytokine production and surface receptor expression in NK cells [9]. At the intracellular level, these flavonoids penetrate cellular membranes to regulate key gene expression networks and functional proteins involved in immune responsiveness [10]. Although direct evidence linking *P. sylvestris* xylem-sap constituents to NK-cell receptor modulation is currently lacking, the documented immunomodulatory activity of plant phenolics provides a plausible basis for investigating whether compounds present in the sap can influence activating receptor expression, cytokine production, and cytotoxic function. Such effects may be mediated through changes in receptor-associated signaling pathways and cellular redox status, providing a mechanistic rationale for integrating antioxidant and immunological measurements in the present study.

Despite the extensive literature documenting the immunomodulatory properties of various terrestrial plant extracts and isolated secondary metabolites on immune cell proliferation and cytotoxicity, the immunological potential of conifer xylem sap remains largely unexplored [11]. Consequently, evaluating how conifer xylem sap influences cell-mediated immune effectors represents an important but understudied area in pharmacognosy.

Understanding these interactions provides valuable baseline insights into NK-cell responses under in vitro conditions. To evaluate these interactions in a standardized and reproducible system, the NK-92 cell line was selected as an established human NK-cell model for studying cytotoxicity, receptor expression, and functional responses. NK-92 cells are highly cytotoxic, IL-2-dependent, CD56-positive, CD2-positive, and lack CD16, thereby providing a relatively defined model for examining NK-cell activation independently of antibody-dependent cellular cytotoxicity (ADCC) [12,13,14]. Standard parental NK-92 cells are interleukin-2 (IL-2)-dependent and lack the CD16 receptor required for ADCC [13,14]. While genetic transfection of CD16 into NK-92 cells has been widely utilized to enhance tumor cytotoxicity and facilitate ADCC mechanisms ex vivo and in vivo [15,16], non-genetic methods to enhance NK functionality remain of broad interest. Multiple Phase I clinical trials using irradiated NK-92 cells have established their safety profile across diverse malignancies, including renal cell carcinoma, lung cancer, melanoma, acute myeloid leukemia, and multiple myeloma [17,18,19,20,21]. Furthermore, ongoing Phase I and II trials continue to evaluate NK-92 platforms in combination with other therapeutics for conditions such as Merkel cell carcinoma, pancreatic cancer, and advanced hematological malignancies [22,23,24]. In the present study, CD56 and CD2 were considered informative lineage/phenotypic markers, whereas CD57 and the activating receptors NKG2D, NKp30, and NKp44 were examined to assess potential sap-induced changes in NK-cell phenotype and activation status. These markers were selected because NK-cell cytotoxicity is closely associated with the coordinated activity of activating receptors, adhesion molecules, and cytotoxic effector pathways [12,13,14]. Therefore, changes in their expression may provide complementary information to functional cytotoxicity and cytokine measurements.

Furthermore, primary peripheral blood lymphocytes from healthy individuals and breast cancer patients were included to complement the standardized NK-92 model with a clinically relevant human cellular context. Breast cancer was selected because impaired NK-cell phenotype and function have been documented in patients with breast cancer, including reduced expression of activating receptors such as NKG2D, NKp30, DNAM-1, and CD16 and reduced cytotoxic activity [25]. Thus, comparison of healthy and breast cancer-derived lymphocytes provides an opportunity to examine whether responses to xylem sap differ according to the immune context of the donor population. Importantly, these primary-cell experiments were intended to complement, rather than replace, the controlled NK-92 model.

Given the limited current information regarding the chemical composition and biological activities of conifer xylem sap, this study investigated *P. sylvestris* xylem sap collected from the Kastamonu region of Türkiye. The primary aim of this work was to characterize the phytochemical profile of *P. sylvestris* xylem sap by determining its total phenolic and total flavonoid contents, and to evaluate its antioxidant potential. In addition, this study evaluated the direct antitumor effect of xylem sap in cancer cell lines, as well as the killing of tumor cells (NK activity) by xylem sap-stimulated NK-92 cells. In this model, NK activity and IFN-γ production were determined by co-culture of primary lymphocytes from healthy individuals and human breast cancer patients with stimulated NK-92 cells. The subpopulations stimulated or suppressed by *P. sylvestris* xylem sap in NK-92 cells were also investigated by immunophenotyping using flow cytometry at baseline and after stimulation. Accordingly, the combined assessment of cell-free antioxidant capacity, direct cancer-cell cytotoxicity, NK-92 immunophenotype, IFN-γ production, and NK-cell-mediated cytotoxicity was designed to provide a multi-level evaluation of the biological activity of *P. sylvestris* xylem sap. This integrated approach allows chemical antioxidant properties to be considered together with direct effects on tumor cells and immune-cell function, thereby addressing an important gap in the current understanding of the biological effects of pine xylem sap. This study aimed to evaluate the in vitro immunomodulatory effects of *P. sylvestris* xylem sap on tumor cells and patient lymphocytes.

## 2. Results and Discussion

### 2.1. LC-MS/MS Profiling of Phenolic Compounds in P. sylvestris

The phenolic composition of *P. sylvestris* xylem sap was characterized by targeted LC-MS/MS analysis following direct extraction and acid hydrolysis. Representative LC-MS/MS chromatograms obtained following direct extraction and acid hydrolysis are provided in the Supplementary Materials (Figures S1 and S2). Analytical validation showed good linearity across the 5–100 ng/mL calibration range (R^2^ > 0.995), with compound-specific LOD and LOQ values of 0.12–0.48 ng/mL and 0.40–1.61 ng/mL, respectively (Table S1). A total of 19 compounds were detected in the directly extracted samples, whereas 18 compounds were quantified after acid hydrolysis (Table 1).

Catechin was the predominant compound detected in the directly extracted xylem sap, reaching a concentration of 2296.69 ± 105.96 µg/g. Other relatively abundant compounds included trans-ferulic acid (43.16 ± 3.69 µg/g), pinocembrin (37.67 ± 3.14 µg/g), phlorizin (21.87 ± 0.91 µg/g), chrysin (14.66 ± 1.48 µg/g), and caffeic acid (14.32 ± 0.31 µg/g). Several additional phenolic and flavonoid-related compounds, including quercetin, luteolin, naringenin, gallocatechin, and epigallocatechin, were also detected.

The qualitative and quantitative phenolic profile obtained here shows both similarities and differences compared with previously reported phytochemical profiles of Pinus species. Phenolic acids and flavonoid-related compounds identified in the present xylem sap, including caffeic acid, p-coumaric acid, trans-ferulic acid, catechin, quercetin, and related flavonoids, have also been reported in different Pinus tissues and extracts. However, the relative abundance of these compounds differs among plant tissues and extraction approaches. In contrast to solid pine tissues, where condensed tannins, stilbenes such as pinosylvin, and resin-related constituents are commonly reported [1,26,27], the present liquid xylem-sap samples contained substantial amounts of relatively low-molecular-weight phenolic and flavonoid compounds. Catechin was the principal compound detected in the directly extracted sap (2296.69 ± 105.96 µg/g), followed by trans-ferulic acid and pinocembrin. This profile is broadly consistent with previous reports showing that phenolic compounds can occur in conifer xylem-associated fluids and tissues, although direct quantitative comparisons should be interpreted cautiously because of differences in species, tissue type, sampling conditions, and analytical procedures [3,4]. The differences observed between direct extraction and acid hydrolysis further suggest that some phenolic constituents may occur in conjugated or otherwise hydrolysable forms, highlighting the importance of extraction and sample-treatment conditions when comparing xylem-sap phytochemical profiles. Notably, catechin decreased from 2296.69 ± 105.96 to 952.77 ± 55.40 µg/g after hydrolysis, whereas epicatechin was not detected in the directly extracted sap but reached 1296.87 ± 159.06 µg/g after hydrolysis. Epigallocatechin, gallocatechin, quercetin, protocatechuic acid, p-coumaric acid, and trans-ferulic acid also showed higher concentrations following hydrolysis. These changes should not be interpreted simply as an increase in the native concentrations of these compounds. Acid treatment can promote the release of phenolic constituents from bound or polymeric forms, while prolonged or strong acidic conditions may also promote degradation, structural rearrangement, or epimerization of flavan-3-ols and related compounds [28,29,30]. Therefore, the increased detection of epicatechin and other phenolics after hydrolysis may reflect, at least in part, the release or transformation of precursor compounds during the hydrolysis procedure rather than their direct accumulation in the native xylem sap.

### 2.2. Total Phenolic and Flavonoid Contents of P. sylvestris

The quantitative analysis of *P. sylvestris* xylem sap showed a substantial presence of phenolic and flavonoid compounds (Table 2). The TPC was recorded at 39.65 ± 2.12 mg gallic acid equivalents per gram of dry lyophilized sap weight (mg GAE/g DW). This substantial phenolic density aligns with the broader understanding of northern coniferous species, which constitutively allocate phenylpropanoid derivatives to vascular tissues for structural maintenance, signaling, and defensive mitigation against abiotic stress factors such as spring frost and thermal fluctuations [31]. Further investigation into the specific polyphenolic distribution revealed a TFC of 21.48 ± 3.11 mg catechin equivalents per gram of dry weight (mg CE/g DW). To express the relative proportion of flavonoids within the total phenolic pool, TPC and TFC values were converted to molar equivalent bases according to their respective standard molecular weights. This calculation revealed that flavonoids account for approximately 54.1% of the total detected phenolic constituents in dry sap. In conifer physiology, xylem sap serves as a dynamic conduit rather than a static transport stream. While solid biomass tissues like outer pine bark typically exhibit much higher absolute phenolic accumulation (>400 mg/g) due to concentrated condensed tannins and lignified matrices [32], the liquid sap matrix maintains a distinct profile. The high relative ratio of flavonoids to total phenolics observed here (54.1%) suggests that the sap is highly enriched with low-molecular-weight, mobile hydrophilic flavonoids (such as catechins, taxifolin, and proanthocyanidin dimers) which are frequently identified as mobile defense compounds in *Pinus* species [33].

### 2.3. Antioxidant Properties of P. sylvestris

To validate whether this distinct phytochemical profile translates into chemical radical-scavenging performance, the antioxidant capacity of the *P. sylvestris* was evaluated across three separate biochemical models (Table 2). The extract demonstrated remarkable uniformity across both radical scavenging assays. The DPPH assay, which evaluates scavenging capacity primarily in lipophilic or organic solutions via Single Electron Transfer (SET), yielded an antioxidant activity of 43.52 ± 2.76 mg TE/g. Concurrently, the ABTS assay, which operates effectively across both aqueous and organic mediums via a combination of Hydrogen Atom Transfer (HAT) and SET mechanisms, returned a highly consistent value of 41.64 ± 2.17 mg TE/g. This tight alignment between DPPH and ABTS radical-quenching metrics indicates that the antioxidant constituents present in the xylem sap are active under different assay conditions.

To complement the radical scavenging data, the FRAP assay was employed to evaluate the sample’s electron-donating capacity to reduce a Fe^3+^-TPTZ complex to its Fe^2+^ form. The sap extract exhibited a robust reducing capability of 39.21 ± 1.95 mg TE/g, showing a strong correlation with both the DPPH and ABTS values. The comparable performance observed across these three cell-free assays suggests that these constituents are capable of efficient electron- and hydrogen-donating reactions. This is supported by the obtained LC-MS/MS results, which revealed the presence of several phenolic and flavonoid-related compounds, including catechin, epicatechin, epigallocatechin, gallocatechin, caffeic acid, trans-ferulic acid, quercetin, luteolin, and pinocembrin. Among these, catechin was the predominant compound detected in the directly extracted sample, while catechin was the predominant compound in the direct extract, whereas epicatechin and epigallocatechin increased markedly following acid hydrolysis (Table 1). Flavans-3-ols and phenolic acids are widely recognized for their radical-scavenging and reducing properties, which may collectively contribute to the antioxidant activity observed in *P. sylvestris* [34,35].

From a mechanistic standpoint, the consistent responses observed across all three assays indicate that the constituents of the xylem sap possess reducing and radical-scavenging properties under varying chemical conditions. The presence of these identified phenolics provides a potential chemical association with the observed antioxidant capacity. Nevertheless, because the assays were performed using the whole xylem sap preparation and individual isolated constituents were not tested independently in single-compound assays, these findings represent a correlative baseline. The observed radical quenching reflects the combined, potential additive or synergistic action of the entire sap matrix rather than proven direct causation by specific individual phenolics. Previous studies have similarly shown that Pinus species contain diverse secondary metabolites with antioxidant properties [36]. However, as these chemical assays evaluate cell-free radical scavenging capacity, they reflect chemical electron/hydrogen transfer properties rather than direct cellular or in vivo antioxidant mechanisms.

It is important to acknowledge that the chemical composition and biological activity of plant secondary metabolites, including those found in xylem sap of *P. sylvestris*, can be significantly influenced by abiotic and biotic factors [1,4,36]. Environmental conditions such as collection site geography, soil composition, seasonal temperature fluctuations, annual precipitation, and tree-to-tree genetic variability are known to modulate metabolic pathways and constituent yields. The xylem sap evaluated in the present study was harvested during a single, defined spring collection period from a specific geographical origin to ensure compositional consistency across all bioassays. While this sample provides a robust and standardized baseline for evaluating the bioactivity of Scots pine sap, it represents a specific spatio-temporal profile. Consequently, future studies incorporating multi-regional sampling across varied microclimates and harvesting seasons will be valuable to evaluate natural compositional variance and establish broader regional standardization.

### 2.4. Changes in NK-92 Cell Surface Receptor Expression Determined by Flow Cytometry

The effects of *P. sylvestris* on NK-92 cell surface receptor expression were evaluated by flow cytometry (Table 3). Compared to the untreated master control, *P. sylvestris* xylem sap decreased CD2 (from 92.24% to 77.83%) and CD7 (from 61.24% to 57.86%) expression, while CD11c, CD45, CD28, and CD56 expression levels remained largely unchanged. In contrast, *P. sylvestris* xylem sap significantly increased CD57 (from 0.15% to 1.05%) and, more importantly, CD16 expression (from 0.00% to 15.93%). The increase in CD16 is of particular interest because CD16 (FcγRIII) is a key receptor involved in ADCC in CD16-expressing NK-cell populations [13,14]. However, increased surface CD16 expression alone does not demonstrate enhanced ADCC, particularly because antibody-dependent target-cell killing was not directly assessed in the present study. Thus, the observed increase should be interpreted as a phenotypic change rather than evidence of increased ADCC function. Further functional studies using antibody-coated target cells would be required to determine whether the observed CD16 upregulation translates into enhanced ADCC.

Regarding the other surface markers, the reduction in CD2 and CD7 and the modest increase in CD57 indicate changes in NK-92 surface phenotype following xylem-sap treatment. CD2 contributes to NK-cell adhesion and co-stimulatory signaling, whereas CD57 is associated with more mature and highly cytotoxic NK-cell subsets [37,38]. Nevertheless, these phenotypic changes should not be interpreted as evidence of a specific differentiation state or increased cytotoxic function without complementary functional measurements.

Because the present study evaluated whole xylem sap rather than isolated compounds or fractions, the observed receptor changes cannot be attributed to a single phytochemical constituent. The LC-MS/MS results demonstrated the presence of multiple phenolic acids and flavonoid-related compounds (Table 1), and interactions among these constituents may contribute to the observed cellular response. However, the specific compound(s) responsible for the receptor modulation remain unidentified and require confirmation through fractionation and targeted functional studies. Previous studies have suggested that dietary polyphenols and flavonoids can influence immune cell function, including NK-cell activity [39,40,41,42,43]. These findings provide a possible context for the present observations, although they do not establish a direct relationship between the individual compounds detected in *P. sylvestris* xylem sap and the receptor changes observed in NK-92 cells.

Overall, the present results indicate that *P. sylvestris* xylem sap can modify selected NK-92 surface markers, most notably CD16. The increase in CD16 is a potentially relevant phenotypic observation, but its functional consequences remain to be established. Further studies incorporating ADCC assays, degranulation measurements, intracellular signaling analyses, and compound-specific testing would be required to determine the underlying mechanism and functional significance of these receptor changes.

### 2.5. Effect of P. sylvestris on IFN-γ Production

Treatment of NK-92 cells with *P. sylvestris* significantly increased IFN-γ production compared with untreated NK-92 cells (Table 4). The concentrations of 1 and 10 µg/mL were selected based on preliminary cell-viability screening, which showed no apparent direct cytotoxicity or loss of NK-92 cell viability during the 24 h treatment period. Basal IFN-γ secretion by untreated NK-92 cells was 11.7 ± 0.85 pg/mL, whereas treatment with *P. sylvestris* increased IFN-γ levels in a concentration-dependent manner to 28.5 ± 1.92 pg/mL (*p* < 0.05) and 33.5 ± 2.10 pg/mL (*p* < 0.01) following treatment with 1 µg/mL and 10 µg/mL, respectively (*n* = 3 independent biological replicates). The increased IFN-γ production observed following *P. sylvestris* xylem sap treatment suggests that the xylem sap enhances NK-92 cell activation.

The increase in IFN-γ production provides evidence of an altered cytokine response following exposure to *P. sylvestris* xylem sap. IFN-γ is an important effector cytokine produced by NK cells and contributes to communication between innate and adaptive immune responses [44]. Similar immunomodulatory effects have been reported for some plant-derived compounds, including curculigoside and oenothein B, which have been associated with increased IFN-γ production in immune cells [45]. However, these previous findings do not establish that the same mechanism is responsible for the response observed with *P. sylvestris* xylem sap. Further mechanistic studies would be required to determine which constituents contribute to the observed increase in IFN-γ production.

### 2.6. Cytotoxicity of P. sylvestris Toward Tumor Cell Lines

**Figure 1:**
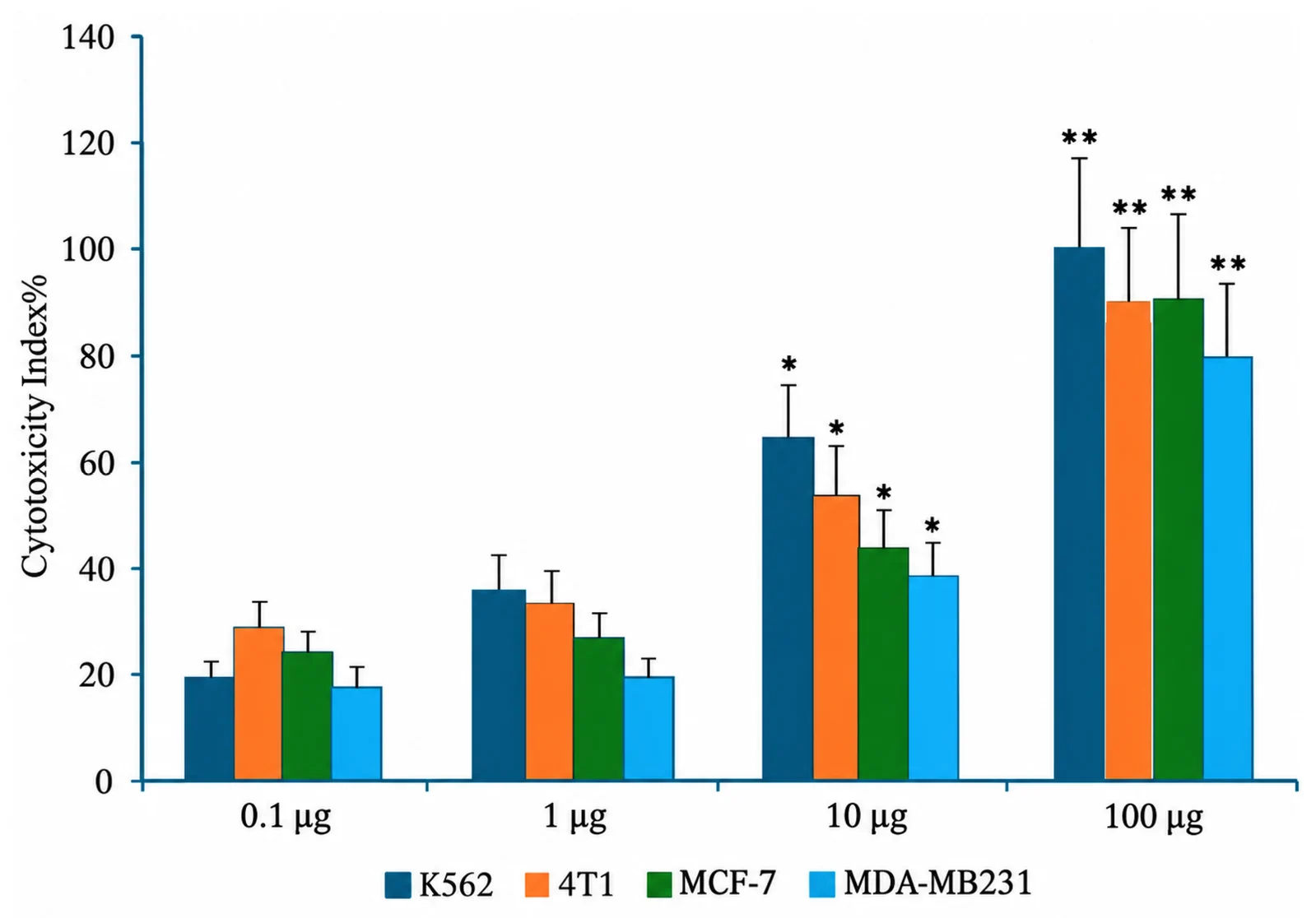
Cytotoxic effects of *P. sylvestris* against K562, 4T1, MCF-7, and MDA-MB-231 tumor cell lines following treatment with increasing concentrations of the extract (*n* = 3 independent biological replicates). * *p* < 0.05, ** *p* < 0.01 vs. untreated control. Doxorubicin was evaluated in parallel as a positive reference control, yielding IC_50_ values of 0.065 µg/mL (K562), 0.152 µg/mL (4T1), 0.190 µg/mL (MCF-7), and 0.245 µg/mL (MDA-MB-231).

**Figure 2:**
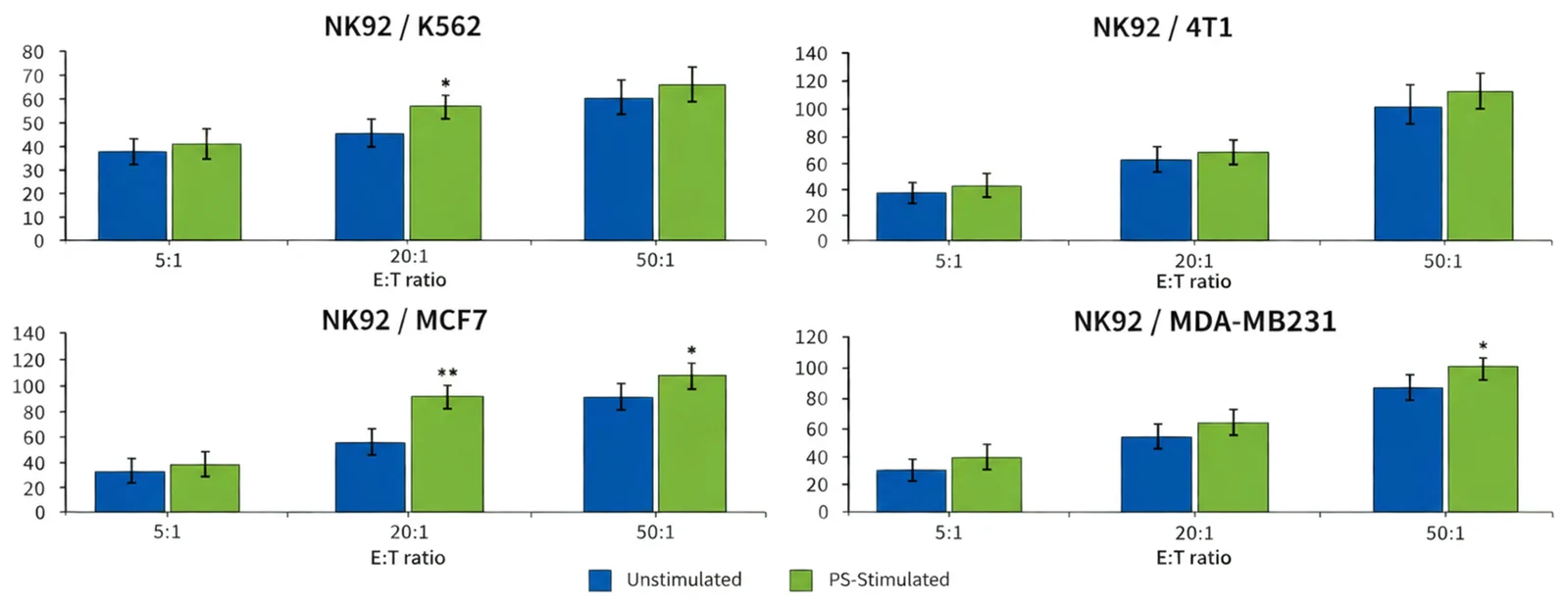
Effect of *P. sylvestris* on NK-92-mediated cytotoxicity against K562, 4T1, MCF-7, and MDA-MB-231 cells at different effector-to-target (E:T) ratios (*n* = 3 independent biological replicates). * *p* < 0.05, ** *p* < 0.01.

The direct cytotoxic effect of *P. sylvestris* was evaluated against K562, 4T1, MCF-7, and MDA-MB-231 tumor cell lines using the MTT assay (Figure 1). The xylem sap exhibited concentration-dependent cytotoxic activity in all tested cell lines (*p* < 0.05). Based on the calculated IC_50_ values, K562 cells were the most sensitive (7.6 µg/mL), followed by 4T1 (9.0 µg/mL), MCF-7 (11.1 µg/mL), and MDA-MB-231 cells (12.5 µg/mL). These concentrations are considerably higher than the concentrations used to stimulate NK activity in NK-92 cells (1 µg). In other words, while *P. sylvestris* is cytotoxic when treated with tumor cells at high concentrations, it modulates the immune system by increasing NK activity at very low, non-cytotoxic concentrations (Figure 2). Although selectivity indices were not determined using non-cancerous epithelial cell lines, subtoxic concentrations (1 µg/mL) exhibited no detrimental effects on primary human lymphocytes isolated from healthy donors, suggesting that low-dose treatments maintain viability in primary immune cells. Future studies evaluating toxicity against non-tumorigenic primary cell models will be required to establish its selectivity index and safety profile.

### 2.7. Effect of P. sylvestris on NK-92-Mediated Tumor Cell Cytotoxicity

After examining the direct toxic effects of *P. sylvestris* on tumor cells (Figure 1), in these experiments, NK-92 cells were incubated in these experiments with *P. sylvestris* at a subtoxic concentration (1 µg/mL) overnight. To determine whether *P. sylvestris* enhances NK cell-mediated cytotoxicity, NK-92 cells were co-cultured with four tumor cell lines at different effector-to-target (E:T) ratios (5:1, 20:1, and 50:1) (Figure 2). In all cell lines, cytotoxic activity increased with higher E:T ratios, indicating a ratio-dependent increase in NK cell-mediated cytotoxicity.

Compared with unstimulated NK-92 cells, *P. sylvestris*-treated NK-92 cells consistently exhibited greater cytotoxic activity against all target cell lines (Figure 2). The most pronounced enhancement was observed against the human breast cancer cell line MCF-7 (*p* < 0.01 at 20:1 and *p* < 0.05 at 50:1 ratio), whereas the smallest increase was detected against the murine breast cancer cell line 4T1. Moderate but consistent increases in NK cell-mediated cytotoxicity were also observed against K562 (*p* < 0.05 at 20:1) and MDA-MB-231 cells (*p* < 0.05 at 50:1). These findings indicate that *P. sylvestris* enhances the natural cytotoxic function of NK-92 cells against both hematological and solid tumor cell targets. The enhanced cytotoxic activity observed following sap treatment is consistent with the literature reports on polyphenol-induced NK cell activation. However, as whole xylem sap contains a mixture of secondary metabolites, these enhanced cytotoxic responses likely reflect multi-component synergistic effects rather than the isolated action of individual phenolics, whose specific identities and direct roles in these pathways remain to be elucidated. For example, pretreatment of SNU-C4 colon cancer cells with quercetin significantly increased their susceptibility to NK-92-mediated lysis [46]. Similarly, Burkard and colleagues [47] reported that myricetin enhanced the cytotoxic activity of NK-92 cells against K562 erythroleukemia cells in a dose-dependent manner across different effector-to-target ratios. These findings support the concept that bioactive phytochemicals can potentiate NK cell-mediated antitumor responses.

Interestingly, the stimulatory effect of *P. sylvestris* was more pronounced against MCF-7 cells than against MDA-MB-231 cells. This observation is in agreement with the findings of Lee & Cho (2020) [48], who reported that MDA-MB-231 cells exhibit greater resistance to NK-92-mediated killing than MCF-7 cells by modulating the expression of activating and inhibitory NK cell receptors and suppressing IFN-γ production. The higher cytotoxicity observed against MCF-7 cells in the present study may therefore reflect differences in the susceptibility of breast cancer cell subtypes to NK cell-mediated immune responses. Flow cytometric analysis confirmed that sap treatment induced statistically significant changes in specific surface receptors across *n* = 3 independent biological replicates (Table 3). Specifically, CD16 increased significantly from 0.00% to 15.93 ± 1.45% (*p* = 0.002), CD57 increased from 0.15 ± 0.03% to 1.05 ± 0.12% (*p* = 0.018), and CD2 decreased from 92.24 ± 2.15% to 77.83 ± 3.12% (*p* = 0.024).

Furthermore, the marked increase in CD16 expression observed in our flow cytometric analysis, together with the enhanced NK-92 cytotoxicity demonstrated in the present study, suggests that *P. sylvestris* may promote phenotypic NK cell priming. However, because specific signaling intermediates were not evaluated, these functional and receptor changes should be viewed as preliminary indicators of immune stimulation rather than a fully characterized activation pathway. Although increased CD16 surface expression was detected, ADCC was not functionally evaluated in this study. Further work is required to determine whether these phenotypic changes translate to functional ADCC in primary cell or complex biological models. Future studies should investigate whether combining *P. sylvestris* with antibody-based immunotherapies can further enhance NK cell-mediated antitumor activity. While these functional cytotoxicity enhancements in NK-92 cells are promising, extrapolation of these in vitro cell-line results to primary human NK cells, complex physiological environments, or clinical oncology requires substantial caution. NK-92 is an immortalized, malignant cell line that depends on exogenous IL-2 and exhibits distinct genetic and phenotypic alterations compared to primary peripheral blood NK cells [49,50]. Primary NK cells possess greater phenotypic heterogeneity, baseline CD16 expression, and complex regulatory receptor networks (such as inhibitory KIRs) that are absent in NK-92 cells. Therefore, these in vitro findings serve as a preliminary proof-of-concept for xylem sap bioactivity rather than clinical evidence of therapeutic efficacy.

### 2.8. NK Cell Cytotoxicity Against Lymphocytes from Healthy Controls and Breast Cancer Patients

**Figure 3:**
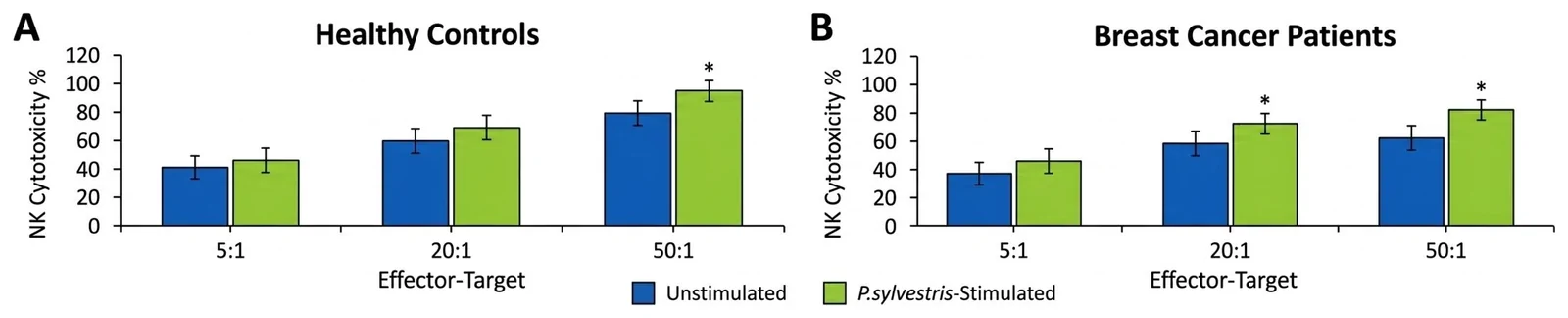
Effect of *P. sylvestris* on NK cell cytotoxicity at different effector-to-target (E) ratios. (**A**) Healthy controls. (**B**) Breast cancer patients. Five independent replicate measurements were performed using the corresponding pooled lymphocyte preparations. * *p* < 0.05.

The effect of *P. sylvestris* on NK cell activity was further evaluated using lymphocytes obtained from healthy individuals (Figure 3A) and breast cancer patients (Figure 3B). The breast cancer patients were 48–65 years old and had disease stages ranging from stage 0 to stage IIB, including Tis, T1bN0, T1cN0, and T2N0–N1a disease. In both groups, stimulation with the xylem sap resulted in higher NK cell cytotoxicity than that observed in unstimulated controls at all effector-to-target ratios. Cytotoxic activity increased progressively with increasing E:T ratios, reaching the highest values at the 50:1 ratio. Although baseline cytotoxicity differed between healthy controls and patients, treatment with *P. sylvestris* consistently enhanced NK cell-mediated killing in both groups, with statistically significant differences observed at 50:1 in healthy controls and at 20:1 and 50:1 in breast cancer patients (*p* < 0.05). Given the limited number of breast cancer donors, these findings should be considered preliminary. Differences in disease stage and other patient-related biological or clinical factors may contribute to variability in lymphocyte and NK-cell responses.

These preliminary in vitro findings indicate that *P. sylvestris* treatment was associated with enhanced cytotoxic activity in lymphocytes obtained from both healthy volunteers and breast cancer patients. The enhanced NK-cell cytotoxicity observed following treatment with *P. sylvestris* may reflect modulation of NK-cell activation; however, the present study did not directly investigate the interaction of individual xylem-sap constituents with specific NK-cell receptors or downstream signaling pathways. Therefore, receptor-mediated activation and its potential contribution to IFN-γ production remain hypothetical. NK-cell cytotoxicity is regulated by the integrated balance of activating and inhibitory receptor signals, and IFN-γ production is influenced by multiple signaling pathways. Accordingly, the present findings provide evidence for an immunomodulatory effect of *P. sylvestris* at the functional level but do not establish a specific receptor-mediated mechanism. Further studies examining NK-cell receptor expression/activation and relevant intracellular signaling pathways are required to clarify the underlying mechanism.

Several limitations of the present in vitro model should be considered when interpreting these findings. First, although NK-92 provides a standardized and reproducible system for evaluating NK-cell responses, it is an immortalized cell line and does not fully reproduce the phenotypic heterogeneity, receptor repertoire, and regulatory properties of primary human NK cells [49,50]. The use of primary lymphocytes from healthy individuals and breast cancer patients partially complements this limitation, but these experiments do not fully reproduce the complexity of the tumor microenvironment. In particular, the present co-culture system does not account for interactions among tumor cells, immune-cell subsets, stromal cells, extracellular matrix components, soluble mediators, and other factors that can influence NK-cell activity in vivo. Second, although changes in surface receptors, IFN-γ production, and cytotoxicity were observed, intracellular signaling pathways and molecular targets responsible for these responses were not directly investigated. Therefore, the observed effects cannot yet be assigned to specific receptor-mediated or downstream signaling mechanisms. Third, the relatively small number of primary donors and the use of a limited number of experimental conditions restrict the generalizability of the findings. Finally, the concentrations tested in vitro cannot be assumed to be achievable or biologically equivalent in vivo, and the pharmacokinetics, bioavailability, metabolism, safety, and tissue distribution of xylem-sap constituents remain unknown. Accordingly, the present results should be considered as preliminary in vitro evidence supporting further mechanistic, pharmacological, and in vivo investigation rather than evidence of clinical efficacy.

In a clinical trial, healthy participant prescribed a diet low in polyphenols and supplemented with juices rich in polyphenols increased lymphocyte proliferative responsiveness, IL-2 secretion and lytic activity by NK cells [51]. An essential consideration in evaluating the translational potential of these findings is the physiological relevance of the concentrations tested. In the present in vitro study, direct cytotoxicity occurred at IC_50_ values of 7.6–12.5 µg/mL, whereas subtoxic immunomodulatory priming occurred at 1 µg/mL. In vivo, systemic bio-accessibility of ingested or administered polyphenols is typically limited by hepatic metabolism, rapid phase II conjugation, and modest plasma peak concentrations (often in the low micromolar or sub-micromolar range) [52,53]. However, because xylem sap is a complex, unrefined mixture, achieving equivalent systemic circulating levels of active constituents would require careful pharmaceutical formulation, such as nano-encapsulation or targeted delivery systems. Consequently, while 1 µg/mL represents a valid subtoxic benchmark for preliminary cell culture models, direct translation to in vivo or clinical therapeutic efficacy must be validated in animal pharmacokinetic and pharmacodynamic models. Furthermore, while our results highlight the cytotoxic and immunomodulatory effects of *P. sylvestris* xylem sap in the context of breast cancer co-culture, future research incorporating dedicated non-tumorigenic cell models is necessary to calculate the exact selectivity index and comprehensively define its therapeutic window.

## 3. Materials and Methods

### 3.1. Collection and Preparation of P. sylvestris Xylem Sap

Xylem sap samples of *P. sylvestris* were collected from three healthy, mature individual trees (*n* = 3 biological replicates) in the Kastamonu region of Türkiye (41°40′26.1″ N, 33°42′25.27″ E; elevation: 1090 m above sea level) in May 2023 during the early spring active sap-flow period. Environmental conditions during sampling were characterized by an average ambient temperature of 14.5 °C, 68% relative humidity, and recent spring snowmelt, which promotes maximal vascular sap pressure and metabolite translocation. Taxonomic identification of the plant species was performed by Dr. Ahmet Beyatli, and a voucher specimen (Voucher No. 240201) was deposited in the Department of Medicinal and Aromatic Plants, University of Health Sciences. The collection procedure was carried out following local traditional practices. Briefly, a vertical strip of the outer bark and phloem was carefully peeled back from the lower trunk to expose the underlying active xylem tissue. Small holes were drilled into the exposed trunk under clean conditions to facilitate sap exudation. The exuding xylem sap was collected directly into sterile containers, immediately kept cold for transport to the laboratory, and stored at −20 °C prior to processing. To prepare the sap for bioactivity testing and phytochemical analysis, the collected liquid xylem sap was frozen and subjected to lyophilization using a lab-scale freeze dryer (Model LBFD-E10, Labtron Equipment Ltd., Camberley, UK) at −50 under reduced pressure until a dry residue was obtained. The resulting lyophilized powder was stored in airtight, light-protected vials at −20 °C until further analysis. The collected xylem sap represents a crude, natural vascular fluid rather than a commercially standardized extract; therefore, laboratory processing (filtration, freezing, and freeze-drying) was strictly standardized to ensure batch consistency for all subsequent assays.

### 3.2. Phytochemical Screening

#### 3.2.1. LC-MS/MS Analysis of Phenolic Compounds

The phenolic profile of *P. sylvestris* was characterized using liquid chromatography–tandem mass spectrometry (LC-MS/MS). Analyses were performed using an Agilent 1260 Infinity liquid chromatography system coupled to an Agilent 6460 Triple Quadrupole mass spectrometer equipped with a Jet Stream electrospray ionization source was used (Agilent Technologies, Inc., Santa Clara, CA, USA). Chromatographic separation was achieved using an Agilent ZORBAX SB-C8 column (3.0 × 150 mm, 3.5 µm) (Agilent Technologies). The mobile phases consisted of 5 mM ammonium acetate in ultrapure water (A) and acetonitrile:methanol (*v*:*v*) containing 0.1% acetic acid (B). The chromatographic separation was performed at a flow rate of 0.70 mL/min and a column temperature of 35 °C using the following gradient program: 0.00 min, 8% B; 0.70 min, 8% B; 3.50 min, 99% B; 8.00 min, 99% B; 8.10 min, 100% B; 9.80 min, 100% B; 10.00 min, 60% B; 11.70 min, 60% B; 11.80 min, 8% B; and 15.00 min, 8% B (re-equilibration).

Two sample preparation procedures, direct extraction and acid hydrolysis, were applied. For direct extraction, 100 µL of *P. sylvestris* was mixed with 900 µL of an extraction solution containing 79% ultrapure water, 20% methanol, and 1% formic acid. After vortexing, sonication at 45 °C for 10 min, and centrifugation at 9000 rpm for 5 min, the supernatant was subjected to LC-MS/MS analysis. For acid hydrolysis, 100 µL of *P. sylvestris* was treated with 200 µL of 2 M HCl and sonicated at 90 °C for 40 min. This acidic treatment was performed to cleave glycosidic linkages and liberate conjugated aglycones; however, potential thermal or acidic degradation of sensitive phenolic structures should be noted when interpreting bound fractions. Subsequently, 700 µL of the extraction solution was added, and the samples were vortexed, centrifuged, and analyzed under the same conditions. The resulting supernatant was used for LC-MS/MS analysis.

Target compounds were identified and quantified using authentic reference standards via dynamic multiple reaction monitoring (dMRM), based on agreement of retention times and precursor-to-product ion transitions with those of the corresponding standards. Quantification was performed using matrix-matched five-point calibration curves prepared at concentrations ranging from 5 to 100 ng/mL, with R^2^ > 0.995 for all analytes. Method performance was evaluated in terms of linearity, precision, recovery, and sensitivity. The method showed acceptable precision (RSD < 10%) and recoveries of 85–115%. The limits of detection (LOD) and quantification (LOQ) were determined using the signal-to-noise approach at S/N ratios of 3:1 and 10:1, respectively, in accordance with ICH Q2(R2) [54]. The LOD values ranged from 0.12 to 0.48 ng/mL, whereas the LOQ values ranged from 0.40 to 1.61 ng/mL. Compound-specific LOD, LOQ, retention time (tR), calibration range, and R^2^ values are provided in the Supplementary Materials (Table S1). Authentic reference standards (≥95% or ≥98% purity, Sigma-Aldrich, St. Louis, MO, USA) used for compound identification and quantification included phenolic acids, flavonoids, flavan-3-ols, stilbenes, and phytohormones (specifically: 2,5-dihydroxybenzoic acid, 2-hydroxytranscinnamic acid, abscisic acid, apigenin, caffeic acid, CAPE, catechin, catechin gallate, chlorogenic acid, chrysin, epicatechin, epicatechin gallate, epigallocatechin, epigallocatechin gallate, ethyl gallate, galangin, gallic acid, gallocatechin, genistein, gibberellic acid, hesperidin, indole-3-acetic acid, isorhamnetin, jaceosidin, jasmonic acid, kaempferol, luteolin, myricetin, naringenin, naringin, oleuropein, p-coumaric acid, phlorizin, pinocembrin, propyl gallate, protocatechuic acid, quercetin, resveratrol, rosmarinic acid, rutin, salicylic acid, sinapic acid, syringic acid, trans-ferulic acid, and verbascoside). Phytochemical profiling and chemical standardization of the unrefined sap were established by quantifying major characteristic marker compounds (e.g., catechin, trans-ferulic acid, and pinocembrin) against five-point matrix-matched calibration curves (*R*^2^ > 0.995) of authentic reference standards.

#### 3.2.2. Total Phenolic Content (TPC) Quantification

The TPC of the extract was quantified using the Folin–Ciocalteu assay. Briefly, each reaction was prepared by mixing 2.5 μL of the extract, 2.5 μL of Folin–Ciocalteu reagent, 195 μL of distilled water, and 50 μL of 7% Na_2_CO_3_. The mixtures were then incubated in the dark for 30 min before measuring their absorbance at 765 nm against a standard curve. This calibration curve was generated using varying concentrations of gallic acid. The TPC of the samples was calculated using the resulting linear equation and expressed as milligrams of gallic acid equivalents per gram of sample [55].

#### 3.2.3. Total Flavonoid Content (TFC) Quantification

The TFC of the samples was determined according to the method described by Dewanto and Colleagues [56]. Briefly, a 0.25 mL aliquot of each extract was mixed with 75 μL of 5% NaNO_2_ and left to react for 5 min. Next, 150 μL of 10% AlCl_3_·6H_2_O was introduced to the mixture, followed by a second 5 min incubation. Finally, the reaction was stopped by adding 0.5 mL of 1 M NaOH, and the absorbance was immediately read at 510 nm. Flavonoid concentrations were calculated using a standard curve and expressed as milligrams of catechin equivalents per gram of sample.

### 3.3. Evaluation of Antioxidant Capacity

#### 3.3.1. DPPH Radical Scavenging Activity

The DPPH radical scavenging potential was assessed using a 0.1 mM methanolic DPPH solution. Briefly, a 100 µL aliquot of the sample was combined with 2.9 mL of the DPPH reagent. The mixture was then incubated in the dark at 25 °C for 30 min, after which the absorbance was recorded at 515 nm. Radical scavenging activity was quantified against a Trolox calibration curve [57].

#### 3.3.2. ABTS Radical Cation Scavenging Assay

For the ABTS assay, the radical cation (ABTS˙^+^) was prepared by mixing 7 mM ABTS with 2.45 mM potassium persulfate. Following appropriate dilution, this stock solution was mixed with the samples at a 1:20 (*v*/*v*) ratio. The decrease in absorbance was monitored at 734 nm after a 6 min incubation period [58].

#### 3.3.3. Ferric Reducing Antioxidant Power (FRAP) Assay

The reducing capacity of the extracts was determined via the FRAP method [58]. The working FRAP reagent was prepared by blending 300 mM acetate buffer (pH 3.6), 10 mM TPTZ, and 20 mM FeCl_3_·6H_2_O. Samples were reacted with 3 mL of this reagent at 37 °C for 10 min, and the absorbance of the resulting Fe^2+^–TPTZ complex was measured at 593 nm. All antioxidant data were expressed as milligrams of Trolox equivalents per gram of extract.

### 3.4. Cell Culture

#### 3.4.1. Cell Lines and Cultures

Human breast carcinoma cell lines (MCF-7 and MDA-MB-231), the murine breast cancer line (4T1), and human erythroleukemia cells (K562) were obtained from the American Type Culture Collection (ATCC, Rockville, MD, USA). K562 cells were cultured in RPMI-1640 medium containing 5% FBS, L-glutamine, and penicillin/streptomycin. MDA-MB-231, 4T1 and MCF-7 cells were cultured under the same conditions but in DMEM medium. NK-92 cells were obtained from the Yeditepe University Genetics Laboratory and cultured in α-MEM medium supplemented with 12.5% fetal bovine serum (FBS), 12.5% horse serum, sodium bicarbonate, and recombinant human IL-2 (10 U/mL). This IL-2 supplementation was maintained consistently across all control and treated culture conditions to preserve baseline viability and NK cell phenotype without inducing vehicle-dependent or variable growth effects.

#### 3.4.2. Cytotoxicity of *P. sylvestris* in Cell Lines

K562, 4T1, MCF-7, and MDA-MB-231 cells were cultured in 96-well plates and treated with *P. sylvestris* extract at serial concentrations of 0.1, 1, 10, and 100 µg/mL. Doxorubicin (0.0054 to 5.44 µg/mL) was included in parallel as a standard positive chemotherapy control. After 72 h of incubation, a 5 mg/mL MTT solution was added to each well. After 4 h of incubation, formazan crystals formed in live cells were dissolved in DMSO, and absorbance was measured at 570 nm using a using a Multiskan GO microplate reader (Thermo Scientific, Waltham, MA, USA) [59]. Cell viability percentages were calculated relative to untreated controls, and IC_50_ values (the concentration required to inhibit 50% of cell viability) were determined by nonlinear regression analysis using a four-parameter logistic curve fit (GraphPad Prism version 8.0).

#### 3.4.3. Stimulation of NK-92 Cells with *P. sylvestris*

Based on the IC50 concentration obtained from tumor cytotoxicity tests, NK-92 cells were incubated overnight (16–18 h) with *P. sylvestris* at a subtoxic concentration (1 µg/mL) and a count of 50,000/mL. The following day, the samples were washed with PBS, centrifuged, and recounted. NK-92 cells, along with primary lymphocytes and target tumor cell lines, were incubated at specific ratios for NK activity assays.

#### 3.4.4. Flow Cytometric Analysis of NK-92 Cell Receptor Expression

Flow cytometry was performed to evaluate the expression of NK cell surface receptors on NK-92 cells following treatment with the extract. The antibody panel was selected to characterize NK-92 cell phenotype and surface receptor expression, including CD2, CD7, CD11c, CD45, CD28, CD56, CD57, and CD16. CD56 was included as an NK-cell-associated marker [49,60], while CD16 (FcγRIII) was evaluated because of its relevance to NK-cell-mediated antibody-dependent cellular cytotoxicity [14,61]. Both stimulated and unstimulated NK-92 cells were analyzed in triplicate (*n* = 3 independent biological replicates) [62]. Cell viability was confirmed to be >95% by trypan blue exclusion before staining.

For each sample, acquisition was performed on a BD FACSCelesta flow cytometer (BD Biosciences, Franklin Lakes, NJ, USA) equipped with 488 nm, 561 nm, and 640 nm lasers. Photomultiplier tube (PMT) voltages were optimized using unstained controls, and single-color compensation beads (BD CompBeads, BD Biosciences, Franklin Lakes, NJ, USA) were used for fluorescence compensation. Cells were gated on forward scatter (FSC-A) versus side scatter (SSC-A) for the lymphocyte population, followed by FSC-H versus FSC-W doublet discrimination. At least 10,000 singlet events were acquired per replicate at <300 events/s. Isotype controls were included to assess non-specific staining and assist in defining negative populations.

Fluorochrome-conjugated monoclonal antibodies targeting CD2, CD7, CD11c, CD45, CD28, CD56, CD57, and CD16 (BioLegend, San Diego, CA, USA) were used for surface-marker analysis. Isotype-matched controls were run in parallel to assess non-specific background fluorescence. The flow-cytometry data were analyzed using FlowJo software (version 10.8.1; BD, Ashland, OR, USA), and the percentage of positive cells was determined for each surface marker.

#### 3.4.5. Effector and Target Cell Assay

Target cells (tumor cells) were prepared at a concentration of 5 × 10^5^ cells/mL and seeded into 96-well U-based plates at various ratios. Effector cells (*P. sylvestris* stimulated NK-92 cells) were co-cultured in triplicate at effector–target (E/T) ratios of 5:1, 20:1, and 50:1. Additionally, 100% tumor cells and 100% NK-92 cells wells were prepared as controls. NK tests were performed with both stimulated and unstimulated NK-92 cells. After incubation at 37 °C with 5% CO_2_ for 24 h, 10 μL of MTT solution was added, and the plates were incubated for another 3 h. Then, 50 μL of supernatant was taken, and 100 μL of SDS was added to dissolve the formazan crystals. After 20 h, absorbance was measured at 570 nm using a microplate reader. NK cell activity was calculated according to a formula described previously [63]:

NK activity (%) = 100% × (ODT − (ODS − ODE))/ODT(1)

In the formula above, ODT is the optical density value of the target cells (tumor cells) control, ODS is the optical density value of the test samples and ODE is the optical density value of the effector cells (NK-92 cells) control.

#### 3.4.6. Co-Culture of NK-92 Cells with Lymphocytes from Breast Cancer Patients and Healthy Subjects

Peripheral blood mononuclear cells were isolated from five breast cancer patients and five healthy volunteers. The breast cancer patients were aged 48–65 years and had disease stages ranging from stage 0 to stage IIB, including Tis, T1bN0, T1cN0, and T2N0–N1a disease. Lymphocyte samples were pooled within each donor group, resulting in one pooled breast cancer lymphocyte sample and one pooled healthy control lymphocyte sample, prior to the co-culture experiments. All participants provided written informed consent, and the study was approved by the Clinical Research Ethics Committee of Istanbul Yeni Yüzyıl University (approval No. 21.09.2021/32).

The pooled lymphocyte samples were subsequently used for co-culture with NK-92 cells at the indicated effector-to-target ratios. For each experimental condition, five replicate measurements were performed independently using the corresponding pooled cell preparation and analyzed separately by flow cytometry. These experiments were performed to evaluate whether *P. sylvestris* could enhance the activity of allogeneic NK-92 cells and promote the destruction of human breast cancer cells.

### 3.5. Gamma Interferon (IFN-γ) Measurement

The NK Vue ELISA kit was used to measure IFN-γ levels. Standards were reconstituted to 4000 pg/mL, and high and low controls were set at 1000 pg/mL and 165 pg/mL, respectively. Serial dilutions were prepared to generate a standard curve (2000–0 pg/mL). Samples and controls were incubated with the detection solution (biotin conjugate and streptavidin-HRP), followed by the TMB substrate. Absorbance was measured at 450 nm. Concentrations were calculated using linear regression (y = ax + b) [64].

### 3.6. Statistical Analysis

All experiments were independently performed in triplicate (*n* = 3 biological replicates), and data are expressed as mean ± standard deviation (SD). For single-factor multiple comparisons (e.g., cytotoxicity assays across concentrations and IFN-γ production), one-way analysis of variance (ANOVA) followed by Tukey’s post hoc test was performed. For two-factor experimental designs (e.g., effector-to-target ratios combined with stimulation status across cell types), two-way ANOVA followed by Sidak’s multiple comparisons test was applied. Flow cytometry receptor expression levels between two groups were compared using Student’s two-tailed *t*-test. All statistical analyses were executed using GraphPad Prism version 8.0 (GraphPad Software, San Diego, CA, USA). Differences were considered statistically significant at *p* < 0.05 and *p* < 0.01.

## 4. Conclusions

In conclusion, this study provides the first comprehensive evaluation of *P. sylvestris* as a potential source of bioactive, immunomodulatory agents. LC-MS/MS analysis revealed the presence of several phenolic and flavonoid-related compounds, providing individual compound-level support for the overall phytochemical profile. Beyond its cell-free chemical radical scavenging capacity and balanced polyphenolic composition, the xylem sap selectively potentiates natural killer cell function at subtoxic concentrations. While chemical assays indicate its radical-quenching capability, further biological studies are warranted to verify its cellular or in vivo antioxidant activity. The observed upregulation of CD16 expression, elevated IFN-γ secretion, and enhanced cytotoxic activity against both solid tumor lines and primary patient lymphocytes demonstrate a marked in vitro immunomodulatory response. However, as the underlying molecular pathways remain to be fully characterized, these findings should be interpreted within the limitations of the in vitro experimental models and should not be considered evidence of therapeutic efficacy. The use of an immortalized NK-92 cell line rather than primary human NK cell subpopulations, alongside the limited number of primary donors and the absence of a physiologically relevant tumor microenvironment, substantially limits the direct clinical translation of these findings. Therefore, further mechanistic, in vivo, pharmacological, and ultimately clinical studies are required to determine the biological relevance and therapeutic potential of *P. sylvestris* xylem sap. Overall, these preliminary cell-line data suggest that the bioactivity and NK-cell-modulating potential of *P. sylvestris* xylem sap warrant mechanistic exploration rather than implying current therapeutic utility or clinical application in oncology.

## Figures and Tables

**Table 1:** Phenolic compounds quantified in *P. sylvestris* by targeted LC-MS/MS following direct extraction and acid hydrolysis.

Compound	Direct Extraction (µg/g)	Acid Hydrolysis (µg/g)
2-Hydroxytranscinnamic acid	9.15 ± 0.74	33.93 ± 2.67
Abscisic acid	3.70 ± 0.20	2.03 ± 0.29
Apigenin	0.39 ± 0.03	ND
CG + ECG	0.17 ± 0.02	ND
Caffeic acid	14.32 ± 0.31	6.82 ± 0.50
Catechin	2296.69 ± 105.96	952.77 ± 55.40
Chrysin	14.66 ± 1.48	ND
Epicatechin	ND	1296.87 ± 159.06
Epigallocatechin	4.43 ± 0.20	269.24 ± 9.66
Gallic acid	0.18 ± 0.02	1.02 ± 0.11
Gallocatechin	5.61 ± 0.44	20.19 ± 0.99
Isorhamnetin	ND	2.94 ± 0.12
Jasmonic acid	1.14 ± 0.08	ND
Luteolin	0.20 ± 0.02	0.79 ± 0.08
Naringenin	10.42 ± 0.14	6.09 ± 0.15
Phlorizin	21.87 ± 0.91	23.94 ± 2.92
Pinocembrin	37.67 ± 3.14	11.96 ± 0.54
Protocatechuic acid	ND	28.86 ± 1.90
Quercetin	1.23 ± 0.05	18.47 ± 0.40
Salicylic acid	1.91 ± 0.09	5.91 ± 0.62
p-Coumaric acid	4.02 ± 0.18	14.89 ± 0.79
trans-Ferulic acid	43.16 ± 3.69	73.97 ± 3.98

ND: Not detected.

**Table 2:** Phytochemical composition and antioxidant activities of *P. sylvestris* (expressed per gram of dry lyophilized sap weight, DW).

TPC (mg GAE/g DW)	TFC (mg Catechin/g DW)	DPPH (mg TE/g DW)	ABTS (mg TE/g DW)	FRAP (mg TE/g DW)
39.65 ± 2.12	21.48 ± 3.11	43.52 ± 2.76	41.64 ± 2.17	39.21 ± 1.95

**Table 3:** Effect of *P. sylvestris* on the expression of NK-92 cell surface receptors determined by flow cytometry (*n* = 3 independent biological replicates). * *p* < 0.05, ** *p* < 0.01.

Surface Receptors	CD2	CD7	CD11c	CD45	CD28	CD56	CD57	CD16
Unstimulated	92.24 ± 2.15	61.24 ± 3.10	99.98 ± 0.02	100.00 ± 0.00	83.27 ± 2.40	99.96 ± 0.04	0.15 ± 0.03	0.00 ± 0.00
*P. sylvestris*-Stimulated	77.83 ± 3.12 *	57.86 ± 2.85	99.94 ± 0.05	100.00 ± 0.00	84.30 ± 1.95	100.00 ± 0.00	1.05 ± 0.12 *	15.93 ± 1.45 **

**Table 4:** Effect of *P. sylvestris* on IFN-γ production by NK-92 cells following treatment with different concentrations of the extract (*n* = 3 independent biological replicates). * *p* < 0.05, ** *p* < 0.01.

Assay	IFN-γ/pg/mL
Unstimulated NK-92 cells	11.7 ± 0.85
NK-92 cells + *P. sylvestris* (1 µg/mL)	28.5 ± 1.92 *
NK-92 cells + *P. sylvestris* (10 µg/mL)	33.5 ± 2.10 **

## Data Availability

The data presented in this study are available within the article.

## Supplementary Materials

The following supporting information can be downloaded at: https://www.mdpi.com/article/10.3390/molecules31203581/s1, Figure S1: LC-MS/MS chromatograms of *Pinus sylvestris* xylem sap following direct extraction; Figure S2: LC-MS/MS chromatograms of *Pinus sylvestris* xylem sap following acid hydrolysis; Table S1: Method performance parameters and calculated limits of detection (LOD) and quantification (LOQ) for targeted LC-MS/MS analysis.
