# Supplementary material for: Immunomodulatory and Antioxidant Activities of Pinus sylvestris L. Xylem Sap: Modulation of NK-92 Cell Receptors and Cytotoxic Function
Source: Molecules. 2026 Oct 9;31(20):3581. doi: 10.3390/molecules31203581 (PMC13707475; doi:10.3390/molecules31203581)
Supplement: Supplementary file 1 [file molecules-31-03581-s001.zip › molecules-4528693-supplementary.pdf]

Immunomodulatory and Antioxidant Activities of *Pinus sylvestris* L. Xylem Sap: Modulation of NK-92 Cell Receptors and Cytotoxic Function

Supplementary Materials

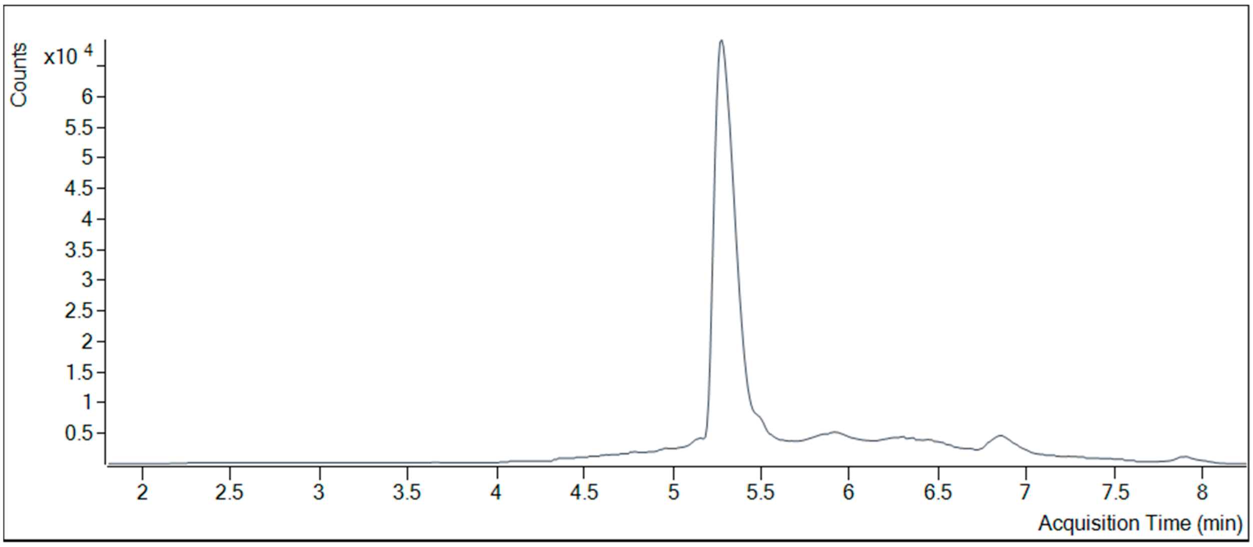

Figure S1. LC-MS/MS chromatograms of *Pinus sylvestris* xylem sap following direct extraction.

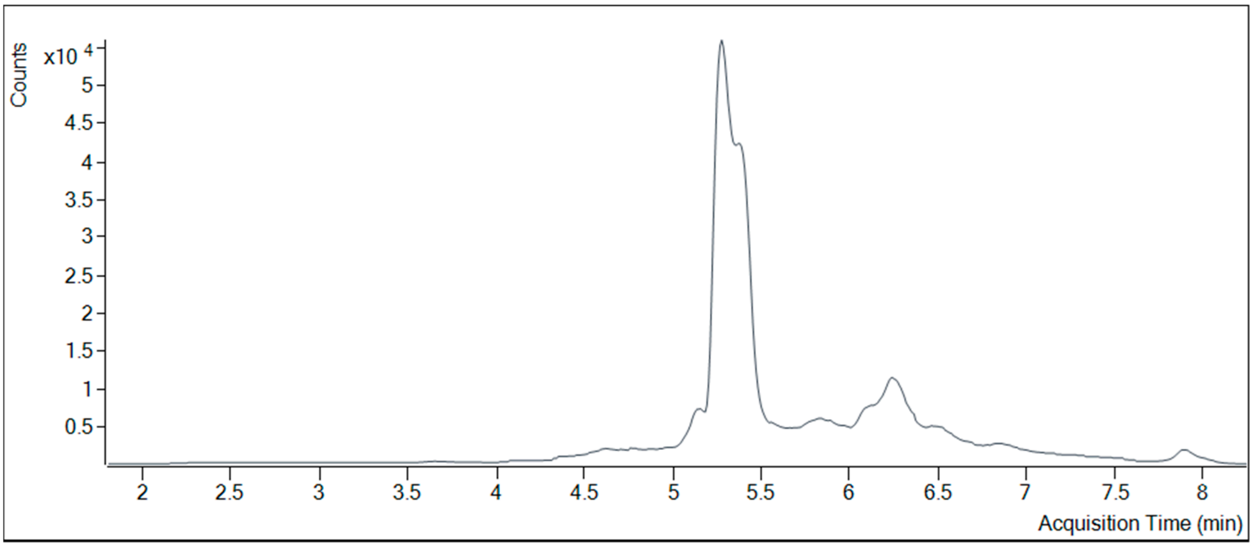

Figure S2. LC-MS/MS chromatograms of *Pinus sylvestris* xylem sap following acid hydrolysis.

Table S1. Method performance parameters and calculated limits of detection (LOD) and quantification (LOQ) for targeted LC-MS/MS analysis.

Compound	Direct Extraction (µg/g)	Acid Hydrolysis (µg/g)	Retention Time (tR, min)	Calibration Range (ng/mL)	R2	LOD (ng/mL)	LOQ (ng/mL)
2-Hydroxytranscinnamic acid	9.15 ± 0.74	33.93 ± 2.67	4.82	5-100	0.9982	0.25	0.83
Abscisic acid	3.70 ± 0.20	2.03 ± 0.29	5.11	5-100	0.9976	0.38	1.26
Apigenin	0.39 ± 0.03	ND	5.64	5-100	0.9991	0.15	0.51
CG + ECG	0.17 ± 0.02	ND	3.95	5-100	0.9968	0.48	1.61
Caffeic acid	14.32 ± 0.31	6.82 ± 0.50	3.42	5-100	0.9989	0.20	0.67
Catechin	2296.69 ± 105.96	952.77 ± 55.40	3.12	5-100	0.9994	0.12	0.40
Chrysin	14.66 ± 1.48	ND	6.21	5-100	0.9979	0.32	1.07
Epicatechin	ND	1296.87 ± 159.06	3.58	5-100	0.9992	0.14	0.46
Epigallocatechin	4.43 ± 0.20	269.24 ± 9.66	2.41	5-100	0.9985	0.22	0.73
Gallic acid	0.18 ± 0.02	1.02 ± 0.11	1.85	5-100	0.9971	0.42	1.41
Gallocatechin	5.61 ± 0.44	20.19 ± 0.99	2.08	5-100	0.9981	0.27	0.90
Isorhamnetin	ND	2.94 ± 0.12	5.78	5-100	0.9988	0.18	0.60
Jasmonic acid	1.14 ± 0.08	ND	5.35	5-100	0.9974	0.40	1.33
Luteolin	0.20 ± 0.02	0.79 ± 0.08	5.28	5-100	0.9990	0.16	0.53
Naringenin	10.42 ± 0.14	6.09 ± 0.15	5.42	5-100	0.9986	0.21	0.70
Phlorizin	21.87 ± 0.91	23.94 ± 2.92	4.15	5-100	0.9983	0.24	0.80
Pinocembrin	37.67 ± 3.14	11.96 ± 0.54	6.05	5-100	0.9987	0.19	0.63
Protocatechuic acid	ND	28.86 ± 1.90	2.75	5-100	0.9978	0.35	1.17
Quercetin	1.23 ± 0.05	18.47 ± 0.40	5.02	5-100	0.9993	0.13	0.43
Salicylic acid	1.91 ± 0.09	5.91 ± 0.62	4.65	5-100	0.9973	0.41	1.37
p-Coumaric acid	4.02 ± 0.18	14.89 ± 0.79	4.10	5-100	0.9984	0.23	0.77
trans-Ferulic acid	43.16 ± 3.69	73.97 ± 3.98	4.38	5-100	0.9989	0.17	0.57

ND: Not detected.
